# Technical, preclinical, and clinical developments of Fc-glycan-specific antibody–drug conjugates

**DOI:** 10.1039/d4md00637b

**Published:** 2024-10-18

**Authors:** Qiang Yang, Yunpeng Liu

**Affiliations:** a Brilliant BioConsultation Ellicott City MD 21043 USA Brilliant.BioConsult@gmail.com; b ChemBind LLC Atlanta GA 30303 USA yliu@chembind.com

## Abstract

Antibody–drug conjugates (ADCs) have emerged as a powerful avenue in the therapeutic treatment of cancer. Site-specific antibody–drug conjugations represent the latest trend in the development of ADCs, addressing the limitations of traditional random conjugation technologies. This article summarizes the innovative development of Fc-glycan-specific ADCs (gsADCs), which utilize the conserved Fc *N*-glycan as the anchor point for site-specific conjugation. This approach offers significant strengths, including improved ADC homogeneity and overall hydrophilicity, enhanced pharmacokinetics and therapeutic index, and potentially reduced Fc receptor-mediated side effects. Currently dozens of gsADCs are in different preclinical and clinical development stages. Notably, JSKN003 and IBI343 have demonstrated promising results in phase 1 trials and are advancing into phase 3 studies. This review discusses the advantages of Fc-glycan-conjugation, various glycan-specific conjugation techniques, and the preclinical and clinical development of gsADCs. While challenges such as increased manufacturing cost for large-scale production need continuous innovation to overcome and there are different opinions regarding the pros and cons of reduced/diminished affinities to Fc gamma receptors, ongoing research and clinical progress underscore the potential of gsADCs to renovate ADC cancer therapy.

## Abstract 2

Fc-glycan-specific ADC is a significant advance in site-specific ADCs for cancer therapy. Notably, JSKN003 and IBI343 have demonstrated promising results in phase 1 clinical trials and are advancing into phase 3 studies.

## Graphical abstract

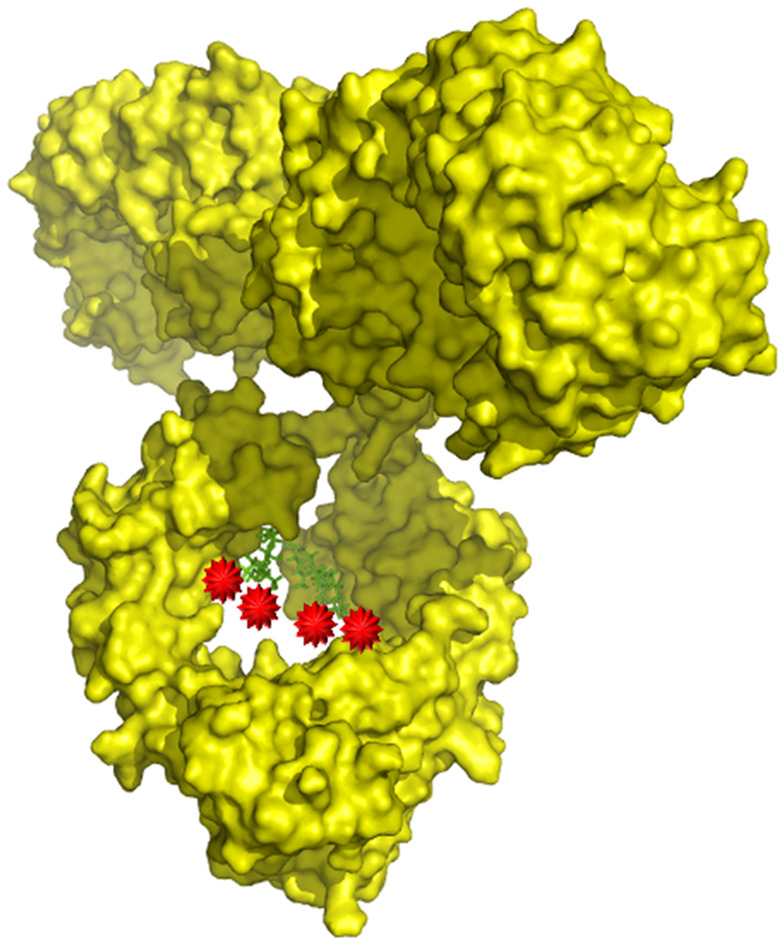

## Introduction

The conjugation of various functional molecules to antibodies is frequently used and actively explored across life science sectors. These conjugates serve diverse purposes, including fluorescently labeled antibodies for detection and imaging,^1^ antibody–drug conjugates (ADCs) for cancer therapy,^2–10^ antibody–antibiotic conjugates for the treatment of infectious diseases,^11,12^ antibody–immunostimulant conjugates for the treatment of cancer and other diseases,^13^ degrader–antibody conjugates (DACs)^14,15^ and LYTAC^16^ for targeted protein degradation. Researchers continue to explore stable, robust, and controlled conjugation technologies in basic research, diagnostics, and therapeutic development, including ADC development.^7,17,18^

The most notable and exemplified application of antibody conjugation is in the development of ADC therapeutics. By attaching a potent cytotoxin to antibodies that specifically binds to cancer-associated antigens, cancer cells are preferentially targeted and killed, while normal cells and tissues remain largely spared. Currently, 15 ADCs have been marketed, with over 200 others undergoing human clinical trials for various types of cancer.^8,9,19^ Many of the ADCs on the market or in trials have payloads conjugated stochastically to either cysteine or lysine residues, resulting in heterogeneous ADC regioisomers with varied antigen affinity, aggregation potential, serum half-life, and other limitations.^7,20–22^ To address these challenges, site-specific ADCs have been developed with more consistent quality attributes (especially drug antibody ratio (DAR)), improved pharmacokinetics, and an enhanced therapeutic index.^7,18^ The majority of explorations and developments to achieve site-specific ADCs have been based on protein engineering, such as engineered cysteines (ThioMab™), peptide tagging followed by enzymatic addition, and unnatural amino acid incorporation.^7,18^ Another option is remodeling of Fc-glycan on the conserved Asn-297 (N297) glycosylation site to generate Fc-glycan-specific ADCs (gsADCs).

**Fig. 1:**
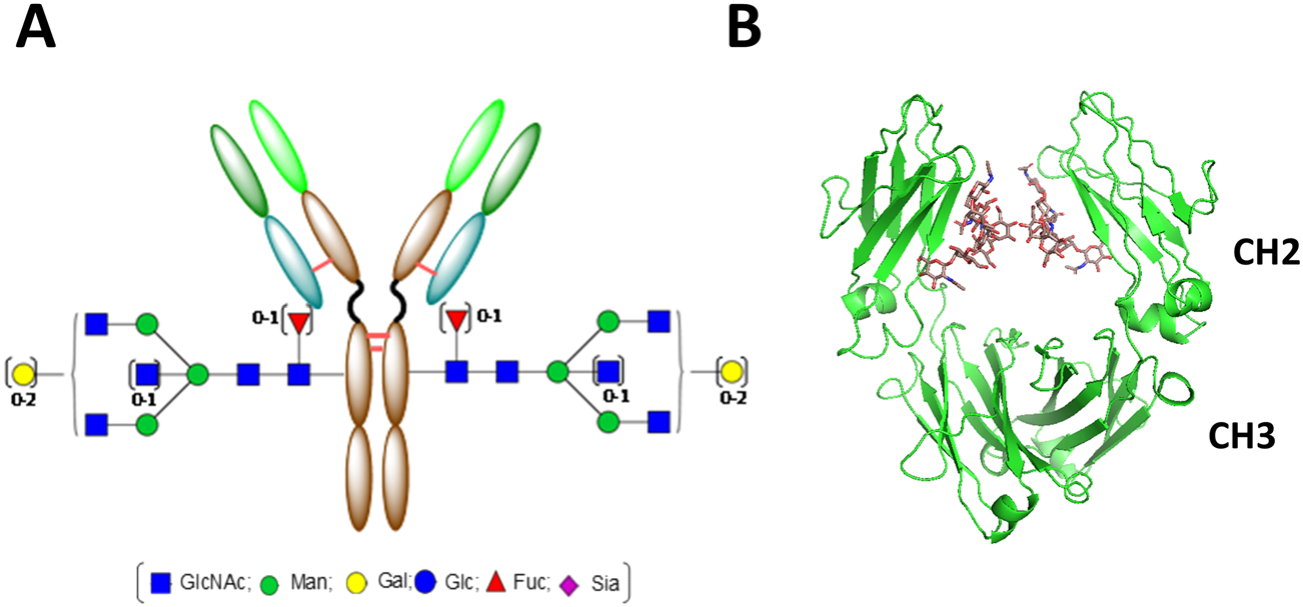
A) Antibody with glycan structure. B) Crystal structure of Fc with G0F glycan (PDB 1H3X).

Glycosylation is the most ubiquitous and diverse posttranslational modification of proteins. It profoundly affects a protein's properties, such as folding, *in vivo* stability, immunogenicity, and pharmacokinetics.^23^ In addition, modification of glycoprotein with natural or synthetic glycan or sugar moieties has provided potential avenues for the development of diagnostic and therapeutic applications.^24–26^ The antibody (more specifically IgG, referred to hereafter if not specified) is the most abundant serum glycoprotein, with a conversed *N*-glycan in the N297 position (Fig. 1A). These *N*-glycans are buried inside a cavity formed by two CH_2_ domains (Fig. 1B) and are predominantly biantennary complex type glycans (Fig. 1A). The monoclonal antibodies produced from CHO cells sometimes contain more than 10% high-mannose glycans while endogenous human IgG1 has less than 1%.^27^ Fc-glycan is crucial for the affinity of the antibody to Fcγ receptors (FcγRs) on the immune cells and the C1q factor of the complementary system, and is therefore critical for the effector functions of the antibodies. If the Fc-glycan of the antibody is removed by mutation of the N297 position, expression in *E. coli*, or enzyme treatment by PNGase F or endoglycosidase, effector functions such as antibody-dependent cytotoxicity (ADCC), antibody-dependent phagocytosis (ADCP), or complement-dependent cytotoxicity (CDC) will be removed or severely impaired.^28^ The contribution of Fc-glycan to the stability of an antibody is less prominent than that of other glycoproteins, such as erythropoietin (EPO), in which the removal of *N*-glycans leads to aggregation and loss of activity.^29^ A PK study of a panel of aglycosylated antibodies (N297A mutation or production in *E. coli*) in cynomolgus monkeys by Leabman *et al.* demonstrated no apparent differences in serum half-life, clearance, or other key PK parameters to their glycosylated comparators.^30^ Although a few studies reported that deglycosylation of antibodies slightly changed the PK, other reports reached a similar conclusion to that of Leabman *et al.*^28,30^ In summary, the data reported so far implies that the role of Fc-glycan in the stability of an antibody in *in vivo* circulation is very limited, for the many antibodies tested. As ADC relies mainly on conjugated drugs as its mode of action (MOA), Fc-glycan becomes a viable choice for the site-specific attachment of payloads. As deglycosylation by PNGase F will facilitate microbial transglutaminase (mTG)-mediated site-specific conjugation to the native Gln-295 (Q295) site, which is very close to the N297 site, several site-specific ADCs generated by mTG technology are also aglycosylated and their effector functions are diminished.^31^

## Fc-glycosylation site N297, an optimal position for site-specific antibody conjugation

**Fig. 2:**
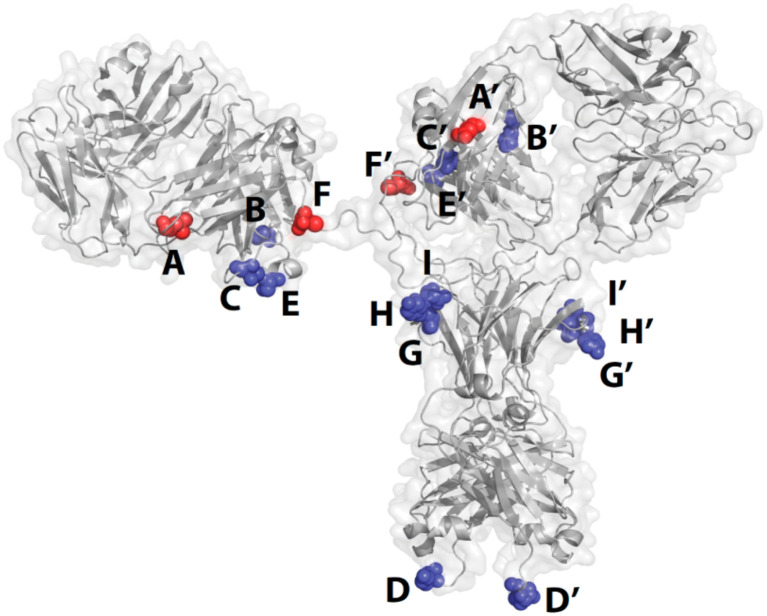
Positional scanning study of site-specific conjugation. Reproduced from ref. 34 with permission from ACS Publications, copyright 2015.

**Fig. 3:**
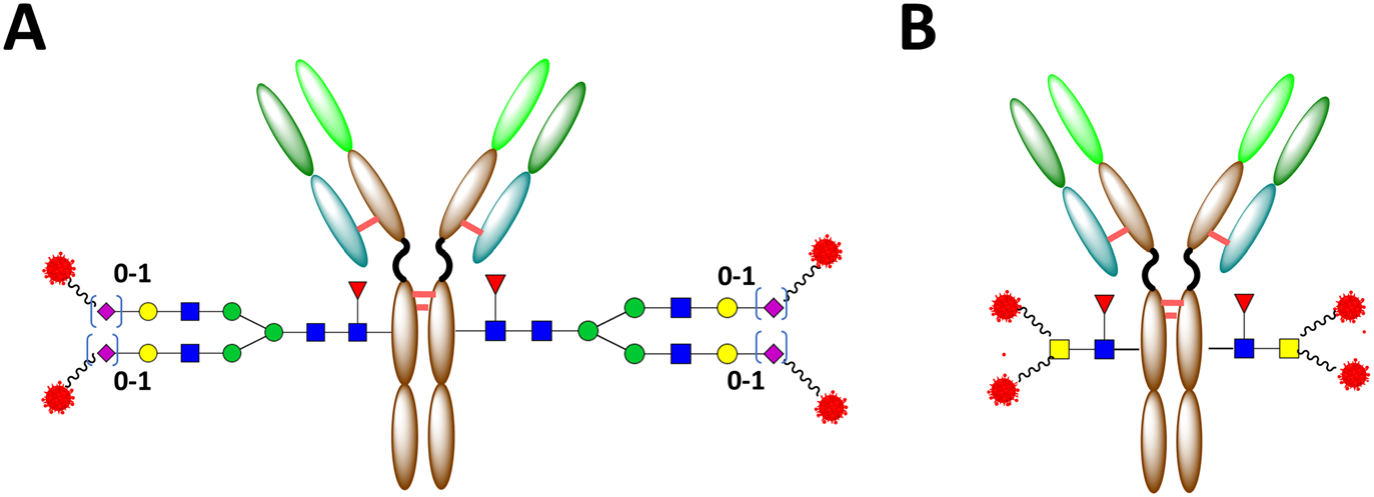
A) gsADC with full-length glycan linkage. B) gsADC with truncated glycan linkage.

Depending on the technological route chosen, linker–payloads will be conjugated to different locations of antibodies, most of them requiring genetic engineering of antibodies. For approaches like SMAC™ or ConjuAll™, where N-terminal or C-terminal tagging is preferred,^32,33^ the location of conjugation is relatively fixed. For engineered cysteine and mTG-mediated conjugation, multiple positions of the antibody can be chosen. Several positional screening studies were carried out with either engineered cysteine or the mTG method. One interesting and imperative finding is that the N297 position and its vicinity appear to be an optimal position for site-specific drug conjugation of an antibody, as demonstrated in multiple preclinical studies and more recently in clinical development. For example, a Pfizer study indicated that among various locations of antibodies (N-terminal of light chain, C-terminal of heavy chain, *etc.*), mTG-catalyzed conjugation on N297Q (H site) or 296Q (I site, Fig. 2), produced cleavable ADC with the highest stability in incubation in mouse plasma, the best pharmacokinetic profile in mice, and the most potent anti-tumor efficacy in a xenograft model.^34^ This resounding observation was corroborated by multiple similar preclinical studies, from Synaffix with GlycoConnect™ technology,^35^ from a Merck research group,^36^ and from researchers at Binghamton University.^37^ The strength and advantages of the Fc-glycan conjugation strategy observed in these studies can be explained by multiple aspects. First, conjugation to the Fc glycosylation is somewhat distant to Fab, thereby not interfering with antigen binding. Second, the natural glycosidic linkage is stable in blood circulation, in contrast to the reversible Michael addition of a cysteine–maleimide connection.^38,39^ Lastly, attachment to a spatially hidden position partially shields the hydrophobicity of the payload and reduces the exposure of cleavable linkers, potentially increasing the overall hydrophilicity of the ADC and reducing the premature release of the payload during circulation. Clinically, two gsADCs have entered phase 3 clinical trials (Fig. 3) while several others are in phase 1 or 2. JSKN003, a bispecific anti-HER2 ADC with a topoisomerase 1 inhibitor (TOP1i) warhead (DAR 4) from Alphamab, utilizes full-length *N*-glycan to connect the payloads (Fig. 3A). The phase 1 clinical trial results for this full-length glycan gsADC are very encouraging, with a favorable tolerability/safety profile and an overall response rate of 56.3%.^40^ With this encouraging result, JNKN003 entered phase 3 trials in December 2023 (NCT06079983). IBI343, an anti-claudin 18.2 ADC from Innovent, is equipped with another TOPi exatecan (DAR 4) with a truncated GlcNAc–GalNAc linkage to the antibody^41^ (Fig. 3B). In a phase 1 trial against gastric/GEJ adenocarcinoma, IBI343 exhibited manageable safety and satisfactory efficacy, with an overall response rate of 32.6%.^42^ Based on these promising results, a phase 3 trial against gastric cancer was initiated in late 2023 (NCT06238843). Multiple other gsADCs, like IBI3001 (B7-H3/EGFR bispecific)^43^ from Innovent, MGC026 (B7-H3 targeting)^44^ and MGC028 (ADAM9 targeting)^45^ from Macrogenics, DS-9606 (claudin 6 targeting)^46^ from Daiichi Sankyo, *etc.*, either with truncated or full-length *N*-glycan linkages, are in early-stage clinical trials, or in preclinical development, and will be thoroughly described in the following section. Collectively, gsADCs represent a promising direction for site-specific ADCs for therapeutic development.

## Various glycan-specific conjugation techniques

**Fig. 4:**
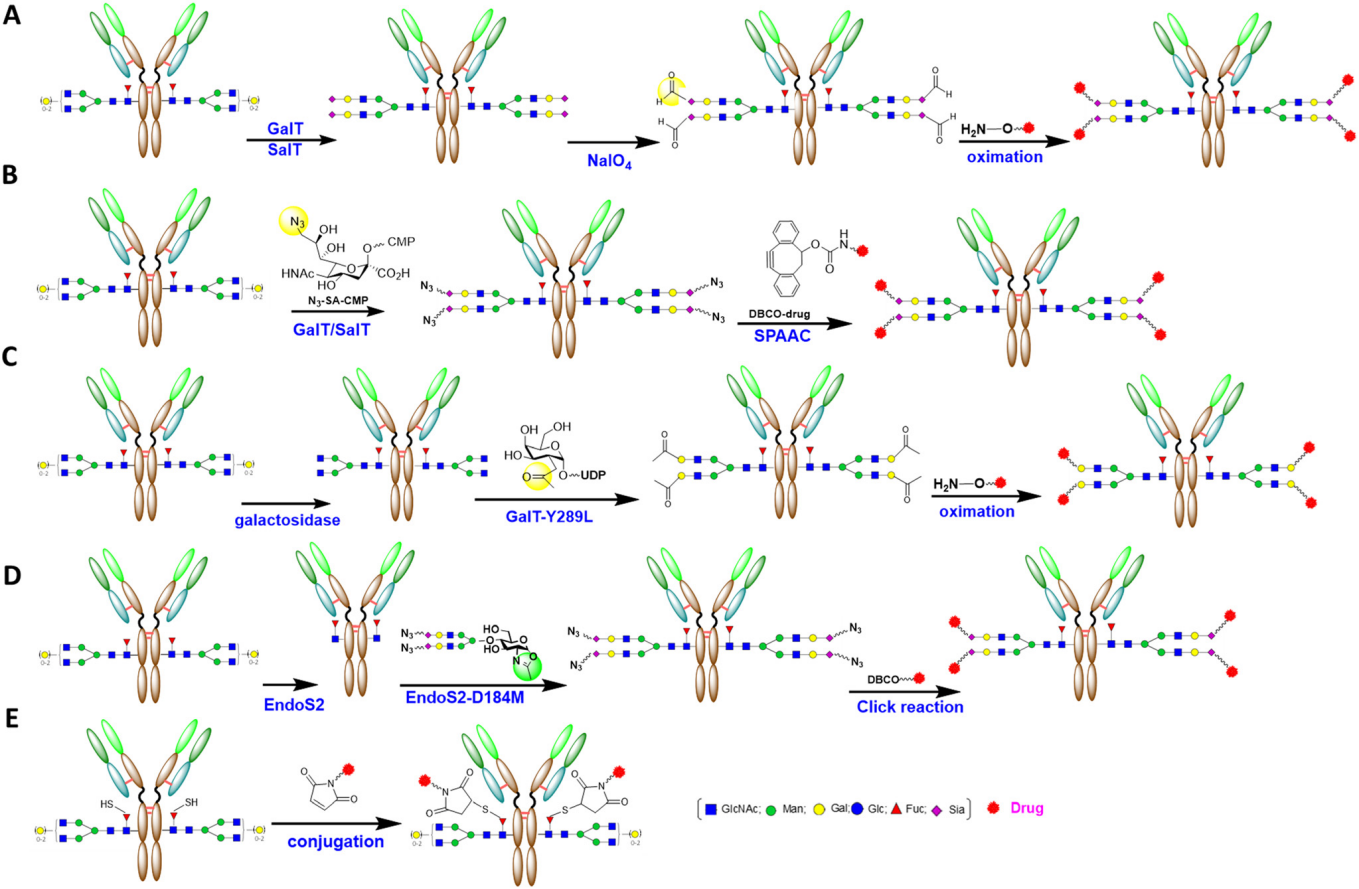
Various methods to prepare gsADCs with a full-length glycan linkage: A) mild oxidation of terminal monosaccharide to introduce a reactive group for linker–payload attachment; B) SalT-catalyzed introduction of Sia-N_3_ followed by SPAAC conjugation; C) GalT-Y289T-catalyzed introduction of Gal-N_3_; D) ENGase-catalyzed *en bloc* transfer of azido full-length glycan; E) metabolic incorporation of 6-thiofucose. Yellow circles indicate active groups for conjugation, while green circles highlight oxazoline groups.

Over the past two decades, multiple approaches have been explored for Fc-glycan site-specific conjugation. Early attempts included the functionalization of Fc-glycans through oxidation of adjacent diols of terminal monosaccharides.^47–50^ This was the first conjugation attempt for the development of gemtuzumab ozogamicin, but oxidation of the antibody lost binding to CD33.^48^ In another successful and representative study by Zhou *et al.*, the antibody was sequentially modified by galactosyltransferase (GalT) and sialyltransferase (SalT) to form a relatively homogeneous sialylated antibody, then mildly oxidized by sodium periodate to form aldehyde groups, and finally conjugated with the payload through oxime ligation^50^ (Fig. 4A). In a separate direction, azide- or keto-tagged sugar nucleotides were probed to introduce an attachable group to Fc-glycan by glycosyltransferase, which is more controllable than oxidation reactions. Li *et al.* pioneered the introduction of azide-functionalized sialic acid onto Fc-glycan with SalT after galactosylation, followed by the conjugation of a dibenzylcyclooctynol (DBCO)-functionalized linker–payload through strain-promoted azide–alkyne cycloaddition (SPAAC)^51^ (Fig. 4B). In another study, Zhu *et al.* utilized an engineered GalT (GalT-Y289L) with enhanced transfer activity to introduce 2-keto-galactose to the terminal of Fc *N*-glycan, which had been degalactosylated by galactosidase. Subsequently, a linker–payload was introduced through an oximation reaction^52^ (Fig. 4C). In a similar manner, azido-galactose was introduced by GalT to a bispecific antibody against HER2, which was further conjugated with DBCO–linker–payload through a SPAAC click reaction, resulting in JSKN003 from Alphamab.^53^ All these methods utilize a combination of exoglycosidase/glycosyltransferase to introduce an attachable group to the Fc-glycan and to achieve homogeneity of the ADC. The resulting ADCs retain full-length *N*-glycan and thus fully or partially retain the antibody effector function. Another choice for preparing full-length gsADC takes advantage of the *en bloc* transfer activity of glycosynthase, which is a mutant of *endo-N*-acetyl-β-d-glucosaminidases (ENGase, a group of endoglycosidases).^54^ As shown in Fig. 4D, ENGase EndoS from *Streptococcus pyogenes*^55^ or EndoS2 from *S. pyogenes* serotype M49 (ref. 56) cleaves Fc *N*-glycan between two GlcNAc of the chitobiose core. Afterwards, the azide, cyclopropene-, or norbornene-tagged sialylated full-length glycan in the activated oxazoline format can be transferred *en bloc* to the GlcNAc-antibody by EndoS-D233Q^57,58^ or EndoS2-D184M^59^*via* a bioorthogonal reaction (Fig. 4D). As EndoS, EndoS2, or their mutants are highly selective to Fc-glycan, their catalyzation of enzymatic glycan remodeling is restricted to Fc-glycosylation, leaving Fab glycan intact.^60^ In addition to using an enzymatic method to introduce conjugational groups, a metabolic engineering method has also been explored. Okeley *et al.* introduced 6-thiofucose to antibody glycan through the metabolic incorporation of a fucose analog, which serves as an anchor point to ligate linker–payloads using maleimide chemistry^61^ (Fig. 4E).

**Fig. 5:**
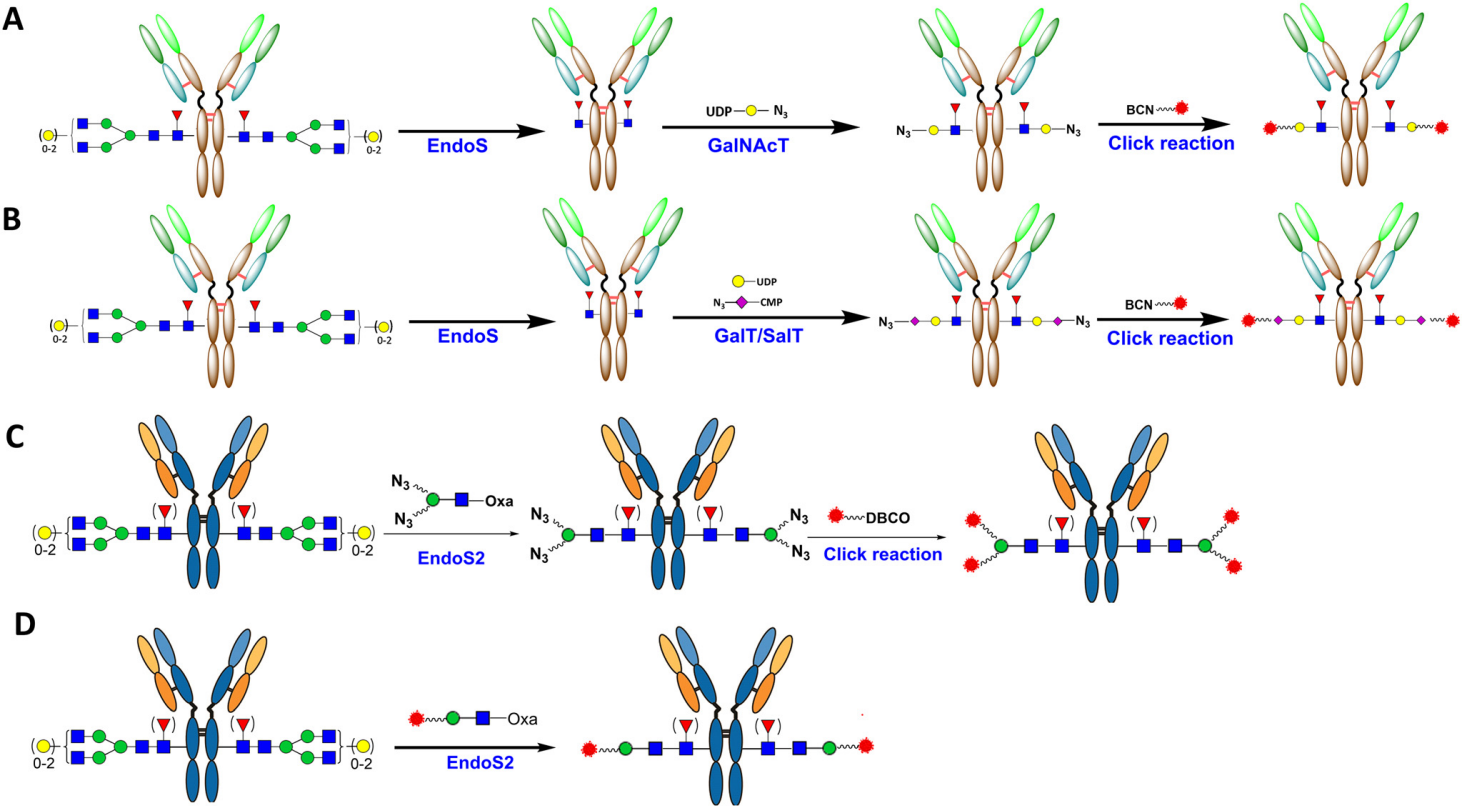
Various methods to prepare gsADCs with a truncated glycan linkage: A) GlycoConnect™ from Synaffix; B) a terminal sialic acid-based gsADC route similar to GlycoConnect™; C) EndoS2-based one-pot glycan-remodeling followed by SPAAC conjugation; D) EndoS2-based one-step gsADC production with preassembled disaccharide–linker–payloads.

In addition to full-length gsADC, gsADC can also be generated with a truncated glycan linkage. An early endeavor was the GlycoConnect™ technology created by Synaffix, which comprises trimming Fc-glycan with EndoS and the subsequent transfer of a GalNAz or an azido-Gal moiety and the final click of the linker–payload (Fig. 5A). Coupled with a variety of linker–payload options, this technology has been used by multiple partnering companies to generate the homogeneous gsADC of DAR 2.^35^ Further improvement in this technology has included the creation of an EndoS–EndoH fusion enzyme to add hydrolase activity to high-mannose glycan (from EndoH) and the introduction of a branched linker to achieve DAR 4.^62^ In a similar technical route, after EndoS trimming and sequential transfer, azido sialic acid was introduced to the terminal of truncated glycan, followed by the click of linker–payload (Fig. 5B).^63^ In another path, taking advantage of the simultaneous deglycosylation/transglycosylation activity of EndoS2, the Wang Lab and Huang Lab established a one-pot glycan remodeling approach, in which the deglycosylation and the transfer of the azido disaccharide to the deglycosylated antibody occur simultaneously in the same reaction system, with just one enzyme^64,65^ (Fig. 5C). As the disaccharide is an unnatural substrate for EndoS2, the transglycosylation product of the azide-functionalized antibody is highly resistant to hydrolysis by the enzyme due to the truncated glycan modification.^64,65^ The azido-antibody can subsequently be conjugated with a DBCO–linker–payload through SPAAC to produce site-specific gsADCs^64,65^ (Fig. 5C). This neat approach shows a clear leading edge towards GlycoConnect™ Synaffix technology, as it reduces the two-enzyme two-step reactions to a single-enzyme one-pot remodeling. In a further exploration, the Huang and Wang Labs have demonstrated that EndoS2 can also accommodate drug-preloaded disaccharide oxazoline derivatives as a substrate for transfer, enabling single-enzyme, one-pot ADC production^65,66^ (Fig. 5D). Nevertheless, the completion and efficiency of such one-pot ADC production is limited to DAR2 ADC and is not optimal or robust, especially for the testing of varied linker–payload combinations or for industrial manufacture. For the Wang Lab and GlycoT (a licensee of Wang Lab technology), in their later report of a scale-up study with different linker–payload combinations, a two-step approach was employed.^19^ A similar issue was also reported in an mTG-mediated site-specific conjugation study, in which a two-step reaction (enzymatic transfer plus click chemistry) produced an ADC with more homogeneous DAR compared to one-step transfer with preassembled linker–payloads.^67^ A two-step approach was employed for the scalable production of an ADC for a PK/PD study due to its better manufacturability.^67^

**Fig. 6:**
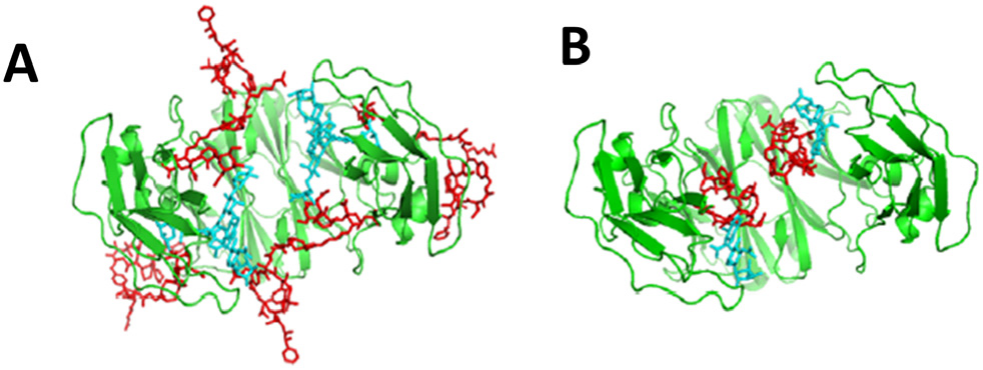
Models of gsADC Fc A) with a full-length *N*-glycan linkage and B) with a truncated glycan linkage. Reproduced from ref. 65 with permission from ACS Publications, copyright 2023.

gsADCs with full-length or truncated linkages are created by different technological routes, and also bear quite different characteristics. Compared to gsADCs with a full-length linkage (FL-gsADCs), the shorter glycan spacer of gsADCs with a truncated linkage (T-gsADCs) may further hide the linker–payloads inside the Fc cavity of antibodies, leading to additionally increased overall hydrophilicity and reduced exposure of linker–payloads to serum protease (Fig. 6A and B). Indeed, in a comparative study of FL-gsADCs *versus* truncated T-gsADCs, the increased exposure of the payload in FT-gsADC was confirmed by a payload-detecting ELISA.^68^ The truncation of *N*-glycan also significantly reduces the ADC affinity to FcγRs, which potentially minimizes the multiple ADC side effects that may be associated with ADC interaction with FcγRs on the immune cells, such as thrombocytopenia^69^ and interstitial lung disease.^70^ The truncation will also diminish the binding of ADC to mannose and asialoglycoprotein receptors, thereby reducing receptor-mediated clearance.^28^ It has been reported that removal of Fc *N*-glycan led to decreased thermal stability and increased potential of pH-induced aggregation for antibodies,^71^ but the addition of linker–payloads into an ADC can compensate for the partial thermal destabilization caused by deglycosylation.^72^ Although truncation of Fc-glycan abolishes ADCC, ADCP and CDC activities (not the main MOA for most ADCs), preclinical and clinical observation from Fc-silenced ADCs prepared with different mechanisms, Fc-glycan truncation,^43–45^ Fc-silencing mutation,^73–76^ and cell-free expression,^77^ implied that such negative potentials were largely very limited or negligible for some or even most ADCs, and such concerns were alleviated at least for multiple clinical stage companies. On the other hand, FL-gsADCs retain affinity with FcγRs and thus with Fc effector-functions. This method may be suitable for antibodies in which ADCC and other effector functions may play a significant role in anti-tumor activity. In addition, it may be well suited to the development of immunostimulatory antibody conjugates.^13^

## Preclinical and clinical development of gsADCs

In the technological development of glycan-specific conjugation approaches, model antibodies like trastuzumab and, less often, brentuximab, have been used to generate gsADCs. These ADCs were benchmarked against FDA-approved stochastic ADC Kadcyla® (T-DM1, with trastuzumab as the mAb component) or Adcetris® (brentuximab as the mAb component) for efficacy and PK/PD profiles. In most cases, gsADCs demonstrated comparable or superior efficacy and PK/PD profiles to their counterparts in rodent models. As a result of this progress, glycan-specific conjugation has been gradually adopted by multiple companies to develop ADC targeting of various tumors. Table 1 summarizes gsADCs in different stages of development.

**Table 1:** gsADCs in different stages of preclinical development

Name	Company	Glyco-conjugation	Target	Linker	Payload (DAR)	Development stage
IBI343	Innovent	GlycoConnect	Claudin18.2	Val-Ala	EXT (4)	Phase 3
IBI3001	Innovent	GlycoConnect	B7-H3/EGFR	Val-Ala	EXT (4)	Phase 1
MGC026	Macrogenics	GlycoConnect	B7-H3	Val-Ala	EXT (4)	Phase 1
MGC028	Macrogenics	GlycoConnect	ADAM9	Val-Ala	EXT (4)	Preclinical
XMT-1660	Mersana Therapeutics	GlycoConnect	B7-H4	Cleavable ester	AF-HPA (DAR 6)	Phase 1
ADCT-601	ADC Therapeutics	GlycoConnect	AXL	Val-Ala	PBD dimer (2)	Phase 1
ADCT-701	ADC Therapeutics	GlycoConnect	DLK-1	Val-Ala	PBD dimer (2)	Phase 1
MRG004A	Miracogen	GlycoConnect	Tissue factor	Val-Cit	MMAE (4)	Phase 1/2
JSKN003	Alphamab	GalT-mediated	HER2 (bispecific)	Gly-Gly-Phe-Gly	TOP1i (4)	Phase 3
JSKN016	Alphamab	GalT-mediated	TROP2/HER3	Gly-Gly-Phe-Gly	TOP1i (4)	Phase 1
DS9606	Daiichi Sankyo	*En bloc* transfer	Claudin 6	Val-Ala	PD (2)	Phase 1
Unnamed	Daiichi Sankyo	*En bloc* transfer	HER2	Val-Ala	CDN (4)	Preclinical

In the progression of glycan-specific antibody conjugation for therapeutic development, Synaffix plays a significant role as a major technology provider and critical driving force in the field. In addition to GlycoConnect™ technology, the company also developed sulfamide-based HydraSpace™ to enhance the overall hydrophilicity of an ADC^78^ and various linker–payload options to provide a one-stop service to ADC companies.^62^ Among the gsADCs developed with Synaffix's technology, IBI343 from Innovent is the front runner in clinical development. It targets claudin 18.2, a tumor associated antigen (TAA) that is overexpressed in gastric, pancreatic, breast and colon tumors and has limited expression in gastric mucosa.^41,79^ IBI343 is armed with exatecan (DAR 4), a TOP1i with a strong by-stander effect. In preclinical evaluation, IBI343 demonstrated excellent stability of the linker–payloads and of the whole molecule in *in vitro* characterization and *in vivo* PK study, impressive *in vivo* efficacy against several cell-line-derived xenograft (CDX) models, and a good safety profile in rhesus monkeys with a highest non severely toxic dose (HNSTD) of 30 mg kg^−1^ in a GLP toxicity study.^41^ In the phase 1a/b clinical study of 159 patients (pts) against advanced gastric/gastroesophageal junction (GEJ) adenocarcinoma, IBI343 exhibited encouraging safety and efficacy profiles. The treatment is well tolerated, with >3 grade treatment related adverse events (TRAE) at 49.7%. Regarding efficacy, for patients with CLDN18.2 expression level at >40%, the objective response rate (ORR) was 32.6%, and the disease control rate (DCR) was 80.9%.^42^ Currently IBI343 has moved to a phase 3 clinical trial towards gastric cancer with a dose of 6 mg kg^−1^ every three weeks (NCT06238843). In another phase 1 clinical trial of 35 pts against pancreatic ductal adenocarcinoma and biliary tract cancer, IBI343 also demonstrated promising results. The safety profile is manageable, with >3 grade TRAE at 37%. In 25 pts eligible for efficacy evaluation, the ORR is 28% and the DCR is 80%. The ORR for pancreatic cancer patients is 40% (4/10) with claudin 18.2 expression level >60%.^80^ Accordingly, IBI343 received fast-track designation by FDA for the treatment of pancreatic cancer in June 2024. In addition to IBI343, another three gsADCs, IBI3001 (B7-H3/EGFR bispecific) also from Innovent,^43^ MGC026 (B7-H3 targeting)^44^ and MGC028 (ADAM9 targeting)^45^ from Macrogenics, were generated with the same Synaffix conjugation and linker–payload technologies and thus bear the same overall structure as IBI343. IBI3001 is built upon a B7-H3/EGFR bispecific antibody (IBI334). It is reported that B7-H3 and EGFR are co-expressed in multiple cancers and dual inhibition of both TAA may provide enhanced treatment outcomes.^43,81,82^ IBI3001 demonstrated better *in vitro* cytotoxicity than B7-H3 ADC or HER3/EGFR bsADC, but the *in vivo* data have not been disclosed. The HNSTD from rhesus monkeys is a remarkable 90 mg kg^−1^, which is just slightly reduced from the 120 mg kg^−1^ of the parent antibody IBI334.^43^ It entered phase 1 clinical trials in early 2024 for multiple solid tumors (NCT06349408). MGC026 from Macrogenics is targeted against B7-H3, a TAA overexpressed in many cancer types.^83^ It demonstrated potent anti-tumor activities in multiple CDX and multiple patient-derived xenograft (PDX) models, with a favorable HNSTD of 50 mg kg^−1^ from a GLP cynomolgus monkey study.^83^ The phase 1 study of MGC026 was initiated in early 2024 (NCT06242470). It is worth noting that MGC026 shares the same antibody component of vobramitamab duocarmazine (MGC018), a duocarmycin-based ADC also developed by Macrogenics. Duocarmycin, a potent DNA alkylator, is at least 10 times more toxic than exatecan.^84,85^ MGC018 is still in phase 2 trials with some setbacks reported recently, including five grade 5 adverse events.^86^ MGC028 is an ADAM9-targeting ADC. It demonstrated potent anti-tumor activities in multiple CDX models, with a favorable HNSTD of 55 mg kg^−1^ from a non-GLP cynomolgus monkey study. MGC028 is in preclinical development.^45^

In addition to the TOP1i payload, GlycoConnect™ technology from Synaffix has also been adopted by another ADC company to develop gsADCs with other linker–payloads. MRG004A, developed by Shanghai Miracogen, is targeted towards TAA tissue factor,^87^ with an MMAE payload (tubulin inhibitor, DAR 4) connected by a cleavable linker.^88^ In a phase 1 clinical trial of 63 pts with multiple solid tumors, MRG004A demonstrated manageable toxicity and encouraging efficacy towards a group of previously heavily treated patients. Most TRAE are grades 1 and 2, but 7.9% (5/63) pts had serious AE (>grade 3). Among 12 evaluable pts, ORR was 33.3% (4/12) and DCR was 83.3% (10/12).^88^ ADCT-601 from ADC Therapeutics, is targeted towards AXL, a tyrosine kinase receptor overexpressed in many solid and hematologic malignancies, and armed with one of most potent payloads, PDB dimer, a DNA alkylator, with a cytotoxicity IC_50_ in the picomolar range.^84,89^ It demonstrated very potent anti-tumor activity towards multiple CDX models and in combination with olaparib, a PARP inhibitor, resulted in synergistic antitumor activity in a BRCA-1 mutated PDX model. The maximum tolerated dose (MTD) of ADCT-601 in rats is 6 mg kg^−1^.^89^ It is notable that rodents are much more tolerant to ADC than non-human primates or humans.^90^ A small clinical trial of ADCT601 against solid tumors was conducted (NCT03700294). Among 8 pts evaluated, one dose-limiting toxicity of grade 3 hematuria was reported, while other toxicities were mild grade 1 or 2. In four pts assessed for efficacy, the ORR was 50%. Afterwards, a large-scale phase 1b study against multiple solid tumors in was launched in 2022 (NCT05389462). In addition to ADCT-601, ADC Therapeutics developed another gsADC ADCT-701 using the same conjugation and linker–payloads that targeted DLK-1 TAA. The ADC showed potent killing in a neuroblastoma CDX model and a hepatocellular carcinoma PDX model and an MTD of 5 mg kg^−1^ in rats.^91^ The phase 1 trial of this ADC against neuroendocrine neoplasms was initiated in 2023 (NCT06041516). XMT-1660, is a gsADC targeting TAA ALX, which is being developed by Mersana Therapeutics with GlycoConnect™ and Mersana's Dolaflexin™ Technology.^92^ The ADC is armed with AF-HPA, a tubulin inhibitor, DAR 6.^93^ In preclinical assessment, this ADC induced tumor eradication in multiple CDX models and anti-tumor activity in a panel of 28 breast cancer PDX.^92^ The phase 1 clinical trial against various solid tumors was launched in 2023 (NCT05377996).

Besides truncated glycan gsADCs, several gsADCs with full-glycan linkage have also moved into clinical development. JSKN003, a bispecific anti-HER2 ADC developed by Alphamab, is armed with TOP1i payloads (DAR 4) that are connected to terminal galactose. The phase 1 clinical trial of 32 pts against various solid tumors with different HER2 expression levels yielded very promising results. The > grade 3 TRAE appeared in 12.5% of pts (4/27), indicating a favorable tolerability and safety profile. As to efficacy, the overall ORR is 56.3% and the disease control rate (DCR) is 90.6%.^40^ With this encouraging result, JNKN003 entered phase 3 trials in December 2023 (NCT06079983). In addition, another bispecific anti-TROP2/HER3 ADC JSKN016 from Alphamab, with a similar ADC structure to that of JSKN003, entered phase 1 clinical trials in early 2024 in China (NCT06592417). Daiichi Sankyo, the leading ADC company, also developed a full-length gsADC DS9606 that targeted claudin 6 TAA. Using a technological route licensed from GlycoT, DS9606 was developed by *en bloc* remodeling of an anti-claudin 6 antibody with a potent pyrrolodiazepine derivative (similar to the PDB dimer, DAR 2) attached to terminal sialic acid.^46^ Daiichi used the EndoS-D233Q/Q303L double mutant, which is reported to retain transglycosylation activity while removing hydrolase activity,^94^ to catalyze the glycan remodeling. Early results of a phase 1 trial of DS9606 with various solid tumors (NCT05394675) were disclosed in September 2024 in an ESMO meeting. 40 pts have been treated while a dose escalation study is ongoing. The safety profile so far is acceptable and four pts achieved a partial response.^95^ In addition to DS9606, Daiichi Sankyo also used the same technology to develop an anti-HER2 immune-stimulating antibody conjugate (ISAC) that is equipped with cyclic dinucleotides (CDN, DAR 4), a STING agonist.^96^

## Limitations and potential solutions

Compared to traditional stochastic conjugation methods, gsADC requires the use of enzyme-catalyzed glycan remodeling to introduce reactive groups for linker–payload conjugation. The addition of the production of enzyme, sugar nucleotide, disaccharide oxazoline, or full-length glycan oxazoline increases the manufacturing cost. The full-length glycan oxazoline is prepared from egg yolk, an animal-derived material, which may complicate the CMC process and add regulatory burden.^97^ Most glycan remodelings need one step of protein A affinity chromatography purification, which further increases the cost of the whole production process.^98,99^ Like other site-specific conjugation technologies, the payloads are concentrated at the anchor point, which may lead to an imbalance in overall hydrophobicity, especially for high-DAR ADCs. Nevertheless, all the above limitations can be addressed by continuous technical innovation and/or be potentially justified by the improved PK and therapeutic index of gsADCs. As has been discussed, the original two-enzyme two-step glycan remodeling of Synaffix can be substituted with a one-enzyme one-pot reaction by the ENGase-based approach, creating an ADC with a similar overall structure. The constant improvement in enzyme manufacture, activated sugar nucleotide, glycan oxazoline synthesis, will greatly reduce the cost of their manufacture. The creation of fusion enzymes for immobilization may eliminate protein A chromatography and streamline the large-scale manufacturing process. The concentration of linker–payload hydrophobicity to the glycosylation site is partially counter-balanced by the hydrophilic glycan linkage and hidden effects from the glycosylation site, and can be further relieved with the introduction of extra hydrophilic groups.^100,101^ The impact of the abolished affinity to FcγRs and thereby diminished effector function of the truncated-linkage gsADC is a topic that still needs further observation and evaluation. Data from preclinical and clinical development so far has shown that the abolished effector function demonstrated no apparent negative effect for the efficacy and *in vivo* stability of gsADCs or other Fc-silenced ADCs, but the putative benefits of reduced side effects and improved PK and therapeutic index need further validation in clinical development.

## Conclusions and outlook

Fc-glycan-specific ADCs are a significant advance in site-specific ADCs for cancer therapy, with the potential for the conjugation of other molecules with different modalities. The launch of phase 3 studies for IBI343 and JSKN003 reflects the accumulation of technical progress and clinical studies of gsADC development over the past decade. Continuous research, technical improvement, and clinical investigations will improve the efficiency of this technical route, overcome current limitations, address the hanging concern for reduced or abolished effector-function, and potentially validate the benefits of Fc-silencing through glycan truncation.

## Data availability

No primary research results, software or code have been included and no new data were generated or analyzed as part of this review.

## Author contributions

Q. Y. and Y. L. write this review and contributed equally.

## Conflicts of interest

Dr. Qiang Yang was the Principal of Brilliant BioConsultation at the time of manuscript submission. Dr. Yunpeng Liu is the Founder of ChemBind LLC.
